# Molecular weight assessment of proteins in total proteome profiles using 1D-PAGE and LC/MS/MS

**DOI:** 10.1186/1477-5956-3-6

**Published:** 2005-06-08

**Authors:** Q Rushdy Ahmad, Dat H Nguyen, Mark A Wingerd, George M Church, Martin A Steffen

**Affiliations:** 1 Dept. of Genetics and Genomics, Boston University School of Medicine, Boston University, 715 Albany St., E639, Boston MA, 02118, USA; 2 Dept. of Genetics, Harvard Medical School, 200 Longwood Ave., Boston, MA 02115, USA; 3 The Lipper Center for Computational Genetics. Harvard Medical School, 200 Longwood Ave., Boston, MA 02115, USA; 4 Dept. of Biomedical Engineering, Boston University, 44 Cummington St., Boston, MA 02215, USA

## Abstract

**Background:**

The observed molecular weight of a protein on a 1D polyacrylamide gel can provide meaningful insight into its biological function. Differences between a protein's observed molecular weight and that predicted by its full length amino acid sequence can be the result of different types of post-translational events, such as alternative splicing (AS), endoproteolytic processing (EPP), and post-translational modifications (PTMs). The characterization of these events is one of the important goals of total proteome profiling (TPP). LC/MS/MS has emerged as one of the primary tools for TPP, but since this method identifies tryptic fragments of proteins, it has not generally been used for large-scale determination of the molecular weight of intact proteins in complex mixtures.

**Results:**

We have developed a set of computational tools for extracting molecular weight information of intact proteins from total proteome profiles in a high throughput manner using 1D-PAGE and LC/MS/MS. We have applied this technology to the proteome profile of a human lymphoblastoid cell line under standard culture conditions. From a total of 1 × 10^7 ^cells, we identified 821 proteins by at least two tryptic peptides. Additionally, these 821 proteins are well-localized on the 1D-SDS gel. 656 proteins (80%) occur in gel slices in which the observed molecular weight of the protein is consistent with its predicted full-length sequence. A total of 165 proteins (20%) are observed to have molecular weights that differ from their predicted full-length sequence. We explore these molecular-weight differences based on existing protein annotation.

**Conclusion:**

We demonstrate that the determination of intact protein molecular weight can be achieved in a high-throughput manner using 1D-PAGE and LC/MS/MS. The ability to determine the molecular weight of intact proteins represents a further step in our ability to characterize gene expression at the protein level. The identification of 165 proteins whose observed molecular weight differs from the molecular weight of the predicted full-length sequence provides another entry point into the high-throughput characterization of protein modification.

## Background

One of the challenges of the post-genome era is the development of technologies and methodologies for the complete characterization of a cell's proteome [1]. This task includes the determination of all protein identities, their amounts, the complexes that they form, their splice forms, and their post-translational modifications. Significant progress has been made on nearly all of these fronts. For instance, protein identities are determined efficiently using 2D-LC/MS/MS [2], or MudPIT [3], or 2DE coupled with MALDI [4]. For the determination of protein quantities, ICAT [5], SILAC [6], and AQUA [7] have made significant contributions. Protein complexes have been characterized in high-throughput fashion using epitope tagging [8,9]. PTMs, in particular phosphorylation, can be targeted using IMAC [10] and other methods [11-13]. Comparatively, there has been relatively little progress with regards to high-throughput characterization of protein splice- or isoforms.

DNA microarray technology revolutionized the field of mRNA profiling [14]. Although mRNA profiling can lend insight into transcriptional control and RNA degradation, it does not directly address translational control of expression, does not characterize PTMs, nor generally identify alternatively spliced transcripts. It is also insensitive to cleavages or chemical modifications of proteins. Since, existing methods for total proteome profiling can, in principle, address many of these issues, there is now a growing need for new tools that can aid in the characterization of these biological processes.

There have been a number of attempts at combining 1D-SDS PAGE with LC/MS/MS for total proteome profiling [15,16]. And there have also been many efforts in which the observed molecular weight of spots on 2D gels are compared to the predicted molecular weight [17,18]. This approach is straightforward and depends on comparison to an external molecular weight marker. While 2D SDS-PAGE is capable of resolving thousands of protein spots, 1D-SDS PAGE offers a number of attractive features, including excellent mass resolution, superior protein solubilization, can accommodate large amounts of protein, and has good run-to-run reproducibility.

In this paper we describe an approach for the automated cataloguing of intact protein molecular weights using 1D-SDS PAGE and LC/MS/MS. This method uses proteins identified in a common gel slice to act as internal standards for each other for the determination of molecular weight of proteins found in that gel slice. We have applied our method to the total proteome profile of lymphoblastoid cells grown on RPI medium.

## Results

### Sample preparation and analysis by mass spectrometry

Lymphoblastoid cells grown in suspension were collected, pelleted and washed, and then lysed by the direct addition of SDS. The total cell lysate was separated on a 16 cm 4–20% gel and stained with Coomassie blue. The entire gel lane was then sliced into 50 fractions, and each was digested manually with trypsin [19]. Peptides were extracted, dried and resuspended in 0.1% formic acid. The fractions were sequentially run on a C18 column with two-hour gradients. Raw data files were analysed with SEQUEST [20]. Fully tryptic peptides which had Xcorr scores that exceeded a threshold (1.75, 2.5, 3.5 for charge states +1,+2,+3, DelCn > 0.1) were compiled.

This procedure identified 1982 proteins (excluding keratins) from 5972 tryptic peptides (see Additional File 1) which differ in their amino acid sequence (hereafter referred to as *unique-sequence *peptides). We then created a subset of that data, requiring that a protein be identified by at least 2 of the above peptides in a single gel-slice fraction. This process did not include those proteins that were identified by two unique-sequence peptides if they were from different gel-slice fractions. This subset of data contained a total of 850 proteins and 4256 unique-sequence peptides, eliminating a total of 1132 proteins and 1716 peptides. All further analyses were performed on the 850 proteins that were identified by at least two unique-sequence peptides in at least one gel slice.

### Method for identification of well-localized proteins

In order to calculate the average molecular weight of proteins within a gel slice, we identified those proteins that migrated as a single well-resolved band in the gel. This was necessary, as we frequently observe that very abundant proteins "smear" along the gel and can be found in all regions of the gel. For example, the worst offender, alpha actin (NP_001091), was observed by at least two unique-sequence peptides in 39 of the 50 gel slices. If actin were included it would distort the average molecular weight calculation in many of the gel slices.

**Figure 1:**
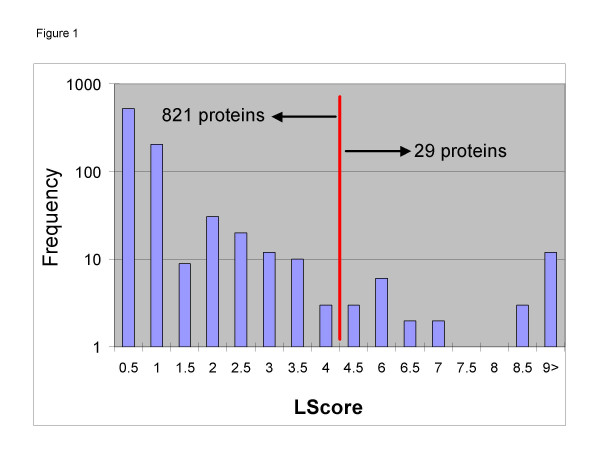
**LScores for observed proteins. **LScores are calculated for each protein based on the gel-slice fractions in which its peptides are observed. A protein that is well-localized and only has peptides in a small number of fractions has a low LScore.

We developed a custom algorithm, called MWFilter [21], to assign a gel localization score, LScore, to each of the 850 proteins. Proteins which migrate as a single well-resolved band have low LScores, and proteins which are smeared out into many fractions have high LScores. LScores are calculated by utilizing the peptide distribution for a given protein, and is the normalized sum of all distances from a peptide hit to the peak of the peptide hit distribution. So, if the *jth *protein has peptide hits in *n *gel slices and the peak of the peptide hit distribution is given by the coordinates (*x*_*p*_, *y*_*p*_) then its localization score is given by the following equation:



If a protein has all its identified peptides in only one fraction then this protein's LScore = 0. For a protein which has peptides in multiple fractions, the algorithm selects the fraction with the greatest number of peptides for that protein, and then calculates the "distance" of all other peptides from that fraction. As another example, actin has an LScore = 45.8. The distribution of LScores for the 850 proteins is shown in Figure 1.

**Figure 2:**
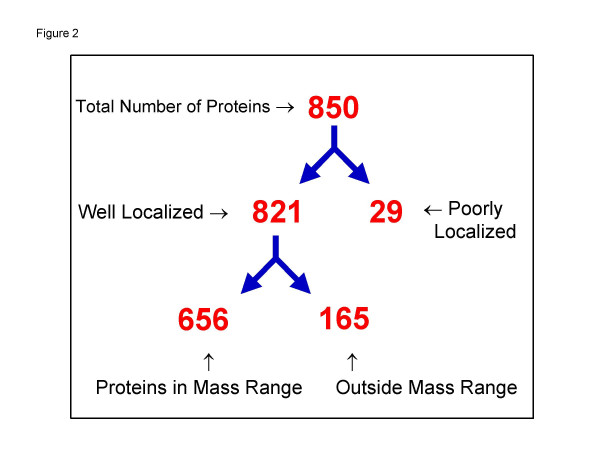
**Summary of total proteome profiling results. **850 proteins were identified by at least two tryptic peptides within one gel-slice fraction. Of these, 821 proteins were localized in the poly-acrylamide gel, while 29 were broadly distributed throughout the gel. These proteins tended to be ones that are generally considered highly-abundant proteins.

Next, we chose an LScore cut-off of one standard deviation away from the mean LScore. This value is 4.25, and separates the 850 proteins into a well-localized group (821 proteins) and a poorly localized group (29 proteins – Figure 2). MWFilter allows the user to specify alternative Lscore cutoff values. We manually inspected the 29 proteins and established that they did appear in multiple fractions spread across the gel.

### Calculation of Average Molecular Weight for each gel slice

**Figure 3:**
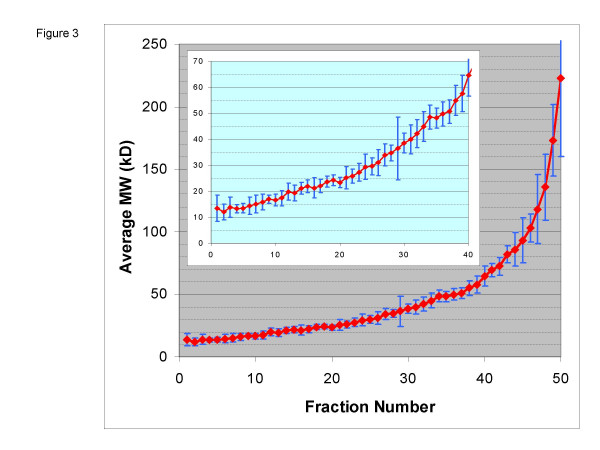
**Molecular weight distribution by gel slice. **The average MW for each gel slice calculated for each gel slice fraction with MWFilter based on the predicted unmodified full-length sequence and plotted in red. The blue bars represent ± 2SD of the molecular weight distribution of the proteins from that fraction (*i.e. *they are *not *error bars, per se). The inset highlights the low MW region of the gel.

The 821 proteins that are well-localized and are identified by at least two peptides in a single gel slice are used to calculate the average molecular weight of proteins within each individual gel slice (MWFilter allows the user to specify the number of peptides required for inclusion in this calculation. If instead the inclusion criteria is three peptides in a gel slice, the calculations are essentially unchanged for this dataset [data not published]). The average molecular weight calculation is performed in two steps. An initial molecular weight distribution is calculated as a means of identifying outliers, which are then removed, and the molecular weight distribution is recalculated in a second step. This sequence of steps was found to be necessary to properly account for modified proteins, and is treated in greater detail in the Discussion section below. Predicted masses for each observed protein were based on unmodified full-length sequences as found in RefSeq. For all proteins observed in a gel-slice fraction, we calculated the average molecular weight (AvgMW) and the standard deviation (StdDev). For the removal of outliers at this stage of the calculation, we removed those proteins whose predicted molecular weight was more than 1 standard deviation from the mean. After removal of the outliers, the AvgMW and StdDev were recalculated, and the results are shown in Fig 3.

Next, for each protein observed in a gel slice, the algorithm compares the predicted full-length molecular weight with the range of molecular weights defined by: AvgMW +/- 2StdDev. If the predicted MW falls within this range, then the protein is scored as being in agreement. If it is outside this range, then the protein is flagged as having a significant molecular weight modification. If a protein, which has already been scored as being well-localized, has at least two peptides in multiple gel slices and is found to match its predicted MW in at least one of these slices, then the protein is considered to be within range. We found for the 821 well-localized proteins, that a total of 656 (80%) proteins showed agreement between their predicted MW and the average MW for that gel slice, and a total of 165 proteins [20%] which had a significant difference between their predicted full-length MW and their location on the gel (Figure 3).

## Discussion

We have developed a software tool for the high-throughput characterization of molecular weights of intact proteins using 1D-PAGE and LC/MS/MS. An observed molecular weight is calculated for a protein based on its location on the gel and the proteins with which it co-migrates. Such an approach is attractive in that it does not require reference to an external standard, or uniform cutting of the gel from one gel to the next. Because of the inevitability of cutting protein bands into multiple gel slices when processing a lane, we devised a score that allows for peptides to be in multiple fractions, while still allowing one to exclude those, primarily abundant, proteins which smear over the entire length of the gel lane. Proteins that are well-localized on the gel and identified by at least two unique-sequence peptides in a given gel-slice fraction act as internal standards for the other proteins in that slice.

The observed molecular weight of a protein can differ from its predicted molecular weight for a number of systematic biological reasons. The mass of a protein can be increased by post-translational modifications, such as glycosylation, ubiquitination, and sumoylation, among others, while the mass can be decreased by alternative splicing and endoproteolytic cleavage. Additionally, there are reports of altered migration for some subsets of proteins, including highly acidic [22], highly basic [23], and arginine-rich proteins [24]. The detailed characterization of these protein-modifying events is one of the goals towards which our MWFilter algorithm strives, yet it also presents a challenge for any algorithm that is in essence a "voting" or "majority rules" type of algorithm. If the majority of proteins in a cell had their molecular weight systematically altered by any mechanism, an average molecular weight of a gel slice calculated from full-length sequences would not be meaningful. However, several lines of evidence indicate that this is not the case, at least in this example. First, as can be seen in figure 2, the majority of proteins, 656 (80%), have observed molecular weights that agree with their predicted molecular weight, based on their unmodified full-length sequence. Secondly, if proteins were significantly modified, it is unlikely that the calculated average molecular weights of each gel slice would be monotonically increasing, as is very nearly the case observed in Figure 3. In this sense, each slice acts as a standard for all other slices. Lastly, calculated molecular weights agree with external standards (data not shown).

In this experiment, we identified 821 proteins that migrate as localized, single bands on a 1D gel. 165 of these proteins, or 20%, have molecular weights that do not fall into the range specified by our algorithm and the proteins with which it co-migrates. 88 of the 165 proteins are observed at lower MW than predicted by the full-length sequence. These proteins are potential candidates for having alternatively spliced transcripts or may be cleaved endoproteolytically. Many proteins in this group are annotated as having signal or transit peptides. If one subtracts the mass due to the signal/transit peptides from the full-length sequence, one observes good agreement between observed and predicted MW (last column, Table 1). Additionally, we observed a total of 77 proteins that have an observed MW that is greater than that predicted by their sequence. PTMs such as glycosylation, ubiquitination and sumoylation can account for reduced migration on gels in principle, but these possibilities need to be investigated by other means.

**Table 1:** Proteins which are potential candidates for endoproteolytic cleavage events.

**Protein ID**	**Protein Name**	**Predicted MW**	**Observed MW**	**Observed Difference**	**Length of Transit/Signal peptide**	**Predicted MW after cleaving of Transit or Signal peptide**	**MW Difference for protein with cleaved leader**
NP_001852	Cytochrome c oxidase subunit IV isoform 1 precursor	19577	16996	-2582	22 AA	17200	-205
NP_002483	NADH dehydrogenase	21750	16666	-5084	46 AA	17000	-334
NP_000088	Coproporphyrinogen oxidase	50175	36543	-13632	131 AA	36900	-357
NP_002114	Major histocompatibility complex, class II, DQ beta 1 precursor	29733	25896	-3837	32 AA	26300	-404
NP_004541	NADH dehydrogenase (ubiquinone) Fe-S protein 2	52545	48185	-4360	33 AA	49000	-815
NP_004083	Mitochondrial short-chain enoyl-coenzyme A hydratase 1	31371	27499	-3872	27 AA	28400	-901
NP_000933	peptidylprolyl isomerase B (cyclophilin B)	23742	19360	-4382	25 AA	20300	-940

A future goal is to extend this method to greater resolution. While 50 fractions per lane represents a practical limit for hand-digestion of gel slices, robots which perform in-gel digestion (*e.g. *Intavis, Cologne, Germany) can extend this number into the hundreds. It is expected that increasing the number of gel-slice fractions will reduce the spread of MW within a slice, thereby allowing the detection of smaller MW changes. These observations will be most useful when comparing a series of related conditions, where "mobility-shifts" of a protein across conditions will highlight functionally relevant changes of a protein's state. Proteins suspected of being alternatively spliced in several conditions can be easily interrogated with RT-PCR, and proteins which are not well-localized only under certain conditions can be examined for the simultaneous presence of multiple isoforms [25]. Additionally, as the analysis of protein complexes using mass spectrometry is an area of increasing interest [2,8,9], this method may be applied to protein complexes separated by native gels.

## Conclusion

We have developed a set of computational tools for extracting molecular weight information of intact proteins in total proteome profiles in a high throughput manner using 1D-PAGE and LC/MS/MS, and applied this method to proteins identified from lymphoblastoid cells. The ability to characterize the molecular weight of intact proteins represents a further step in our ability to characterize gene expression at the protein level. All 50 gel slices in our experiment were assigned an average MW and corresponding StdDev, which were then used to determine the observed MW of a given protein. We identified 165 proteins (20%) that have molecular weights that differ from their predicted full-length sequence. These 165 proteins are likely to be enriched for proteins whose MW has been altered by an interesting biological process, such as alternative splicing, endoproteolytic processing, and post-translational modifications. As such, MWFilter provides a convenient entry point for the discovery and characterization of protein processing events.

## Methods

### Sample Preparation

Cells were grown in suspension to early stationary phase in Iscove's media containing 10% fetal calf serum and pen-strep in 5% CO_2 _at 37°. Cells were pelleted in a 50 ml conical tube, washed three times with PBS, and lysed by the direct addition of gel-loading buffer containing 2% SDS. The sample was sonicated to reduce viscosity. Proteins were separated on a 16 cm, 4–20% polyacrylamide gel (Jules Inc., Milford, CT) and visualized by Coomassie staining. The entire gel lane was manually cut into 50 sections, and subjected to in-gel tryptic digestion [19].

### Mass spectrometry

An aliquot of each fraction was injected onto a C18 reverse phase column using a ThermoAS autosampler with Surveyor pumps (ThermoFinnigan, San Jose, CA). Nanospray columns were constructed by packing a 10 cm bed of MAGIC C18 AQ reverse phase bulk media (Michrom Inc.; Auburn, CA) into pulled, fritless 75 micron ID fused silica capillaries under pressure. Gradients were from 0%-30% B buffer in 90 minutes, followed by 30%-90% B in 10 minutes (Buffer A: 0.1% formic acid; Buffer B: 0.1% formic acid in acetonitrile). The nanospray column was directly interfaced to the orifice of an LTQ ProteomeX ion trap mass spectrometer (ThermoFinnigan) and mass spectra were recorded. From a single parent scan (MS) spectrum, the ten most abundant ions were selected for collision-induced dissociation (CID). MS^2 ^spectra were collected for each of these top ten ions. If a particular parent ion was observed more than 3 times in a 2 minute span, it was excluded from analysis for the subsequent 3 minutes (dynamic exclusion). Mass spectra were analyzed by SEQUEST [20]. Fully tryptic peptides with a SEQUEST XCorr score of > 1.75 (Z = 1), 2.5 (Z = 2), and 3.5 (Z = 3), and DeltaCn >0.1 were queried against RefSeq entries that have index numbers of the form NP_XXXXXX.

## Competing interests

The author(s) declare that they have no competing interests.

## Authors' contributions

QRA performed sample preparation, analysis and wrote software. DN aided in algorithm development. MAW assisted in mass spec analysis. MAS and GMC participated in the design and coordination of the study.

## Supplementary Material

Additional File 1 MultiConsensus Data file Click here for file
